# Early retinal microvascular abnormalities in patients with chronic kidney disease

**DOI:** 10.1111/micc.12555

**Published:** 2019-05-27

**Authors:** Ling Yeung, I‐Wen Wu, Chi‐Chin Sun, Chun‐Fu Liu, Shin‐Yi Chen, Chung‐Hsin Tseng, Hsin‐Chin Lee, Chin‐Chan Lee

**Affiliations:** ^1^ Department of Ophthalmology Keelung Chang Gung Memorial Hospital Keelung Taiwan; ^2^ College of Medicine Chang Gung University Taoyuan Taiwan; ^3^ Department of Nephrology Keelung Chang Gung Memorial Hospital Keelung Taiwan; ^4^ Community Medicine Research Center Keelung Chang Gung Memorial Hospital Keelung Taiwan; ^5^ Department of Chinese Medicine Chang Gung University Taoyuan Taiwan

**Keywords:** capillary, chronic kidney disease, macula, microvasculature, optical coherence tomography angiography, retina, vessel density

## Abstract

**Objective:**

To evaluate early retinal microvascular abnormalities in patients with chronic kidney disease (CKD) via optical coherence tomography angiography.

**Methods:**

A cross‐sectional study. Two hundred patients with CKD stage ≧3 were enrolled in the CKD group, and 50 age‐matched healthy subjects were enrolled in the control group. Main outcome measures were the differences in parafoveal vessel densities in the superficial vascular plexus (SVP) and deep vascular plexus (DVP) between the CKD and control groups.

**Results:**

The mean ages were 62.7 ± 10.1 in the CKD group and 61.9 ± 9.7 (*P* = 0.622) in the control group. The CKD group had reduced parafoveal vessel densities in SVP (46.7 ± 4.3 vs 49.7 ± 2.9, *P* < 0.001) and DVP (50.1 ± 4.1 vs 52. 6 ± 2.9, *P* < 0.001) when compared to those of the control group. In multiple linear regression models, age, diabetes, estimated glomerular filtration rate, and use of anti‐hypertensive drugs were factors associated with vessel density in SVP, whereas age, diabetes, and smoking were factors associated with vessel density in DVP.

**Conclusion:**

Patients with CKD had reduced vessel densities in parafoveal SVP and DVP, as compared to that of control subjects. Microvasculature in the different retinal layers may be affected by different systemic factors.

**Abbreviations**
ACEI: angiotensin‐converting enzyme inhibitors
AMD: age‐related macular degeneration
ARB: angiotensin receptor blockers
BCVA: best‐corrected visual acuity
BMI: body mass index
BP: blood pressure
CKD: chronic kidney disease
DM: diabetes mellitus
DVP: deep vascular plexus
eGFR: estimated glomerular filtration rate
FAZ: foveal avascular zone
FD‐300: foveal vessel density in 300‐μm‐wide region around FAZ
LogMAR: logarithm of the minimum angle of resolution
OCTA: optical coherence tomography angiography
RAS: renin‐angiotensin system
SD: standard deviation
SVP: superficial vascular plexus

## 1 INTRODUCTION

Chronic kidney disease is a common comorbidity in ophthalmologic patients, especially among old aged, hypertensive, and diabetic patients. The prevalence is estimated to be 8%‐16%^1^ and increases with age. It may be as high as 30.8% at age 70 or more.^2^ It can also be found in 15% of non‐diabetic hypertensive patients^3^ and in 43%‐53% of patients with diabetes.^4^ The prevalence may double by 2035 with the anticipated increase in diabetic and older population.^5^ Chronic kidney disease has been associated with accelerated atherosclerosis, cognitive impairment, cerebrovascular disease, cardiovascular disease, and mortality.^6^, ^7^, ^8^, ^9^ In the eye, patients with CKD have higher risks of cataract, glaucoma, AMD, retinopathies, and visual impairment.^10^, ^11^, ^12^ The mechanism behind increased ocular diseases in patient with CKD is still being debated. It may be due to CKD and ocular diseases sharing many common systemic risk factors such as aging, DM, hypertension, smoking, and obesity.^11^ Alternatively, it could also be due to mechanisms related to CKD, such as increased oxidative stress by decreased filtration of free radical‐generating nitrogenous waste products or increased inflammation by activation of the RAS.^11^

Earlier studies have revealed that decreased retinal vessel caliber, smaller fractal dimensions, focal arteriolar narrowing, arteriovenous nicking, and opacification of the arteriolar wall can be found in patients with CKD.^13^, ^14^, ^15^ These retinal microvascular changes may be useful biomarkers for predicting cardiovascular diseases,^16^ cognitive impairment,^17^ and aggravation of renal function in patients with CKD.^18^, ^19^ However, there is limited information about microvascular alterations at the capillary level.^20^ Although increased intercapillary distance in CKD has been shown through use of scanning laser Doppler flowmetry,^20^ the changes in different layers of the retinal vascular plexus are unknown. It is also unclear what role other systemic comorbidities play in these microvascular changes.

Optical coherence tomography angiography is a newly developed non‐invasive diagnostic tool that provides a depth‐resolved three‐dimensional image to visualize the different layers of the retinal vascular plexuses. The purpose of this study was to evaluate early retinal microvascular changes in the superficial and DVPs in patients with CKD through use of OCTA. We also evaluated systemic factors associated with these changes. Elucidating these retinal microvascular changes may (a) shed some light on the pathogenesis of ocular diseases in CKD; (b) help interpret retinal OCTA images in patients with CKD; and (c) provide information for future pharmacological intervention to improve visual outcomes.

## 2 MATERIALS AND METHODS

This single‐center, cross‐sectional study was conducted between August 2017 and July 2018 by the Department of Nephrology and the Department of Ophthalmology at Keelung Chang Gung Memorial Hospital, Keelung, Taiwan. The study was approved by the Chang Gung Memorial Hospital Institutional Review Board, and it followed the tenets of the Declaration of Helsinki. The inclusion criteria for the CKD group were (a) age ≧21 years; (b) CKD stages 3‐5 (including end‐stage renal disease); and (c) no visual symptoms. The inclusion criteria for the control group were (a) age ≧21 years; (b) no major systemic disease; (c) no visual symptoms; and (d) no retinal and macular diseases. The exclusion criteria were (a) the presence of significant ocular media opacity (such as dense cataract); (b) inability to obtain adequate quality OCTA image (scan quality score <6/10 or presence of significant artifact); or (c) pregnancy.

Chronic kidney disease was defined, using the criteria recommended by Kidney Disease: Improving Global Outcomes (KDIGO) 2012 Clinical Practice Guidelines, as (a) abnormalities of kidney structure or function, present for >3 months, with implications for health; and (b) decreased glomerular filtration rate to <60 mL/min/1.73 m^2^ for >3 months.^21^ Estimated glomerular filtration rate was calculated from serum creatinine concentration using the CKD Epidemiology Collaboration equation.^22^ Severity of CKD was defined by eGFR categories: 30‐59 mL/min/1.73 m^2^ (stage 3), 15‐29 mL/min/1.73 m^2^ (stage 4), and <15 mL/min/1.73 m^2^ (stage 5).^23^ Patients with CKD meeting the above criteria were enrolled from the Department of Nephrology. Age‐matched (same age‐group) healthy subjects without retinal disease were enrolled into the control group in 4:1 ratio. An informed consent was obtained from each subject.

Medical histories and laboratory data for the most recent 3 months were gathered. Medical history was collected through a standardized questionnaire and electronic medical records. Taiwan's National Health Insurance provides a nationwide electronic platform that allows for the sharing of patients’ medical information, prescription records, laboratory data, and other examination reports with the patients’ informed consent. Subjects suspected to have major systemic diseases were excluded from the control group.

The BMI was calculated from measured weight and height. Complete ocular examinations, including BCVA, intraocular pressure, slit‐lamp biomicroscopy examination, indirect fundus ophthalmoscopy, color fundus photographs, axial length, optical coherence tomography, and OCTA, were performed. Best‐corrected visual acuity was measured on a Snellen chart and converted to the logMAR for calculation. The presence of any retinopathy was documented. Early AMD (AREDS category 2) is characterized by multiple small drusen (<63 μm in diameter), few intermediate drusen (63‐124 μm in diameter), or mild retinal pigment epithelial abnormalities.^24^ Diabetic retinopathy was classified via the International Clinical Diabetic Retinopathy and Diabetic Macular Edema Disease Severity Scales.^25^ In patients with both eyes eligible, the eye with better OCTA quality was used for statistical analysis.

### 2.1 Optical coherence tomography angiography parameters

AngioVue (Optovue RTVue XR Avanti; Optovue Inc.) was used for acquiring OCTA images for this study. The machine uses an 840‐nm diode laser source and has an A‐scan rate of 70 kHz. A 3 × 3‐mm scan, centered on the fovea, was performed in all eyes. An orthogonal registration algorithm was used to produce a 3‐dimensional OCTA image. Then using the machine's AngioVue software (version: A2017,1,0,151), the vascular area was automatically segmented into four layers, that is superficial, deep, outer retina, and choroidal. The default segmentation for the SVP includes vasculature between the internal limiting membrane and 10 μm above the inner plexiform layer. For the DVP, this includes the vasculature between 10 μm above the inner plexiform layer and 10 μm below the outer plexiform layer.

**Figure 1:**
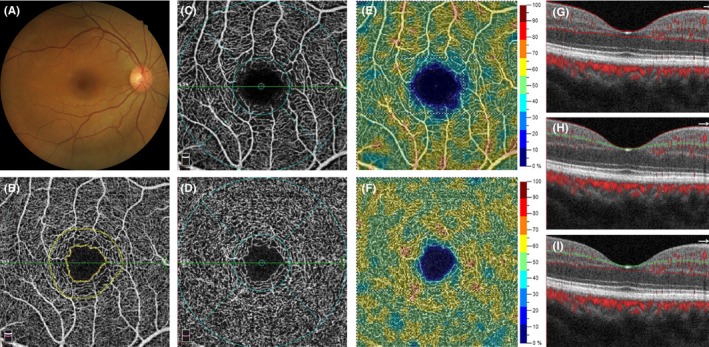
A 49‐y‐old healthy woman in the control group. (A) Normal color fundus photograph. (B) An OCTA image of the inner retina, which contains of both the SVP and DVP. Inner yellow line demarcates the boundary of FAZ. The inner and outer yellow lines demarcate the 300‐μm‐wide region around the FAZ. (C) An OCTA image of the SVP. The blue colored grid is an ETDRS grid that contains the foveal region in a 1‐mm‐diameter circle and the parafoveal region within a 1‐mm‐wide circular annulus. (D) An OCTA image of DVP. (E) An vessel density map of SVP. (F) An vessel density map of DVP. (G) A B‐scan image shows the segmentation site at the inner retina (between the two red lines), located at the green line in (B). (H) A B‐scan image shows the segmentation site at SVP (between the red and green lines), located at the green line in (C). (I) A B‐scan image shows the segmentation site at DVP (between the green and red lines), located at the green line in (D)

The vessel density is defined as the percentage area occupied by all vessels (including terminal arterioles, venules, and capillaries) in a particular region. The data are provided in an ETDRS grid vessel density map (Figure 1). The foveal region is a 1‐mm‐diameter circle, and the parafoveal region is a 1‐mm‐wide circular annulus. The parafoveal region was further divided into the temporal, superior, nasal, and inferior quadrants. The AngioVue software automatically calculates the vessel density of the SVP and the DVP, respectively. We also evaluated other foveal parameters provided by the machine software including the FAZ size; FAZ perimeter; FAZ a‐circularity index; and FD‐300. The foveal parameters were determined from an OCTA image of the inner retina microvasculature, which contained both SVP and DVP (Figure 1).

### 2.2 Statistical analysis

To compare the demographic data and clinical characteristics of the CKD group with the control group, Pearson's chi‐square test was used for categorical variables and the independent sample *t* test was used for continuous variables. The independent sample *t* test was also used to analyze differences in vessel densities and foveal parameters between the two groups. Multiple linear regression models with backward stepwise method were used to determine the potential systemic factors associated with the vessel densities in SVP and DVP of all subjects (Model 1) and of patients with CKD (Model 2). Age, sex, smoking status, BMI, DM, use of anti‐hypertensive drugs, systolic BP, diastolic BP, CKD, CKD stage, and eGFR were independent variables entered into the regression models whenever applicable. A two‐tailed *P* value <0.05 was considered as statistically significant. Data were analyzed using SPSS Program Package version 17.0 (SPSS Inc.).

## 3 RESULTS

**Figure 2:**
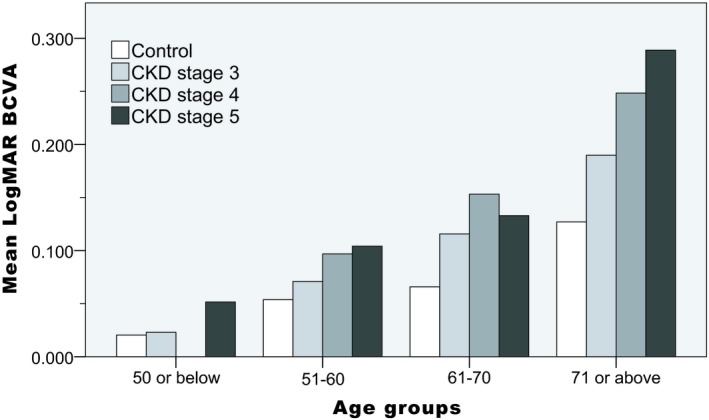
The distribution of mean logMAR BCVA in the control and CKD groups

There were 200 patients enrolled in the CKD group and 50 healthy subjects enrolled in the control group. The mean age was 62.7, SD (±) 10.1, in the CKD group, and 61.9 ± 9.7 in the control group (*P* = 0.622). The demographic data and clinical characteristics are summarized in Table 1. There were no significant differences in age‐group, sex, diastolic BP, smoking status, cerebrovascular disease, intraocular pressure, or axial length between two groups. However, the mean BCVA in patients with CKD (logMAR: 0.130 ± 0.151, Snellen equivalent 20/27) was slightly worse than that of the control group (logMAR: 0.069 ± 0.103, Snellen equivalent 20/23) (*P* = 0.001). Figure 2 shows the mean logMAR BCVA in different stages of CKD. There is a trend toward worse visual acuity with more severe CKD.

**Table 1:** Demographic data and clinical characteristics

	Control group (n = 50)	CKD group (n = 200)	*P* value[Link]
Age (mean ± SD)	61.9 ± 9.7	62.7 ± 10.1	0.622
Age‐group, n (%)			
50 or below	7 (14.0)	29 (14.5)	0.996
51‐60	11 (22.0)	41 (20.5)	
61‐70	22 (44.0)	89 (44.5)	
71 or above	10 (20.0)	41 (20.5)	
Sex, n (%)			
Female	27 (54)	81 (40.5)	0.085
Male	23 (46)	119 (59.5)	
BMI, mean ± SD	23.8 ± 3.1	25.7 ± 5.0	0.001
Systolic BP (mm Hg), mean ± SD	131 ± 15	139 ± 20	0.009
Diastolic BP (mm Hg), mean ± SD	75 ± 9	77 ± 13	0.215
Smoking, n (%)	5 (10)	27 (13.5)	0.508
DM, n (%)	0 (0)	91 (45.5)	<0.001
Use of anti‐hypertensive drug(s), n (%)	0 (0)	176 (88)	<0.001
Cardiovascular disease, n (%)	0 (0)	40 (20)	0.001
Cerebrovascular disease, n (%)	0 (0)	2 (1.1)	1.000
LogMAR BCVA (mean ± SD)	0.069 ± 0.103	0.130 ± 0.151	0.001
Intraocular pressure, mm Hg (mean ± SD)	14.9 ± 2.3	15.0 ± 2.7	0.762
Axial length, mm (mean ± SD)	24.19 ± 1.21	23.88 ± 1.35	0.131

Comparing the CKD group and control group, the *P* values were calculated via independent sample *t* test for continuous variables and chi‐square test for categorical variables.

Chronic kidney disease group also had significantly higher value in BMI, systolic BP, prevalence of DM, number of patients using anti‐hypertensive drug, and prevalence of cardiovascular disease. The systemic conditions and classes of anti‐hypertensive drugs in CKD group are summarized in Table 2. There were 116 (66%) patients who used more than one class of drugs.

**Table 2:** Systemic conditions and classes of ant‐hypertensive drugs using in patients with CKD patients

Systemic conditions in 200 CKD patients	
**Etiology of CKD, n (%)**	
DM	75 (37.5)
Hypertension	49 (24.5)
Gout	11 (5.5)
Other systemic diseases	7 (3.5)
Chronic glomerulonephritis	25 (12.5)
Polycystic kidney disease	9 (4.5)
Other renal or urinary tract diseases	11 (5.5)
Unknown etiology	13 (6.5)
**CKD stage, n (%)**	
Stage 3	81 (40.5)
Stage 4	43 (21.5)
Stage 5	76 (38.0)
**Treatments, n (%)**	
Hemodialysis	27 (13.5)
Peritoneal dialysis	33 (16.5)
Kidney transplantation	3 (1.5)
Creatinine (mg/dL), mean ± SD	4.68 ± 4.37
eGFR (mL/min/1.73 m^2^), mean ± SD	26.9 ± 19.8

Classes of drug in 176 CKD patients using anti‐hypertensive drug(s), n (%)	
ACEI/ARB	128 (73)
Calcium channel blockers	87 (49)
Beta‐blockers	75 (43)
Diuretics	51 (29)
Alpha‐1 blockers	17 (10)
Vasodilators	9 (5)
Alpha‐2 agonists	2 (1)

**Figure 3:**
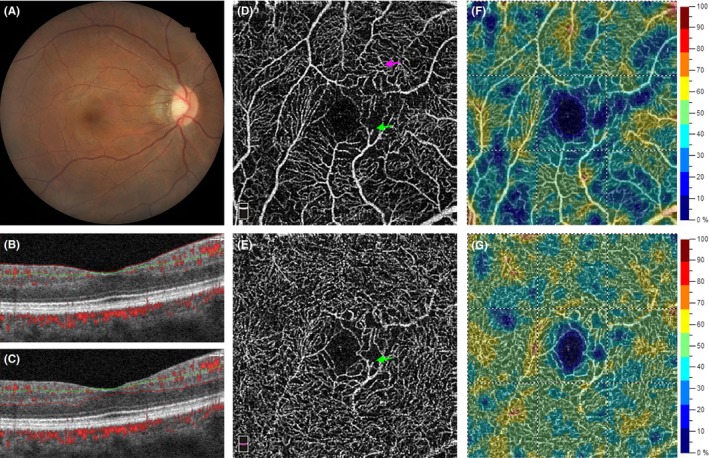
A 41‐y‐old male patient with CKD stage 3. (A) Color fundus photograph reveals mild attenuation of retinal arteries. (B) A B‐scan image shows the segmentation site at the SVP and (C) at the DVP. (D) An OCTA image of SVP and (E) of DVP. The green arrows indicate a blunt‐ended retinal vessel. The purple arrow indicates the area with increased vessel tortuosity. A localized non‐perfusion area can be found at the nasal side of the FAZ. (F) A vessel density map of SVP. Multiple areas of capillary rarefaction are shown in deep blue. (G) A vessel density map of DVP. A few areas of capillary rarefaction can be found at the nasal and temporal‐superior side of the FAZ

**Figure 4:**
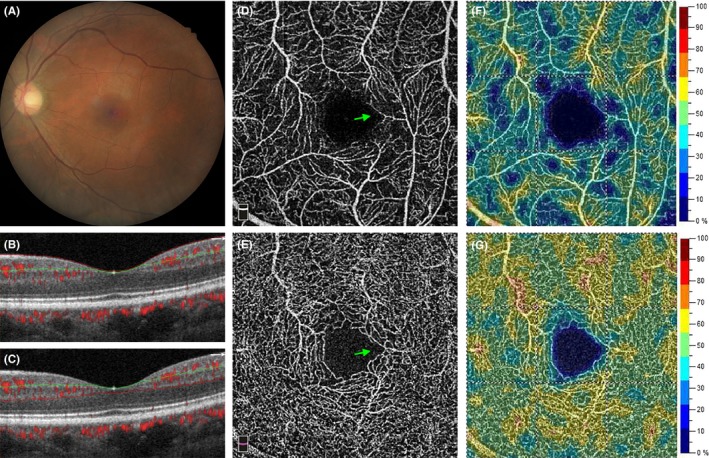
A 48‐y‐old male patient with CKDstage 5. (A) Color fundus photograph reveals mild attenuation of retinal arteries. (B) A B‐scan image shows the segmentation site at the SVP and (C) at the DVP. (D) An OCTA image of SVP and (E) of DVP. The green arrows indicate a disruption of the parafoveal capillary at SVP and DVP. (F) A vessel density map of SVP. Multiple areas of capillary rarefaction are shown in deep blue. (G) A vessel density map of DVP. DVP vessel density was well preserved in this patient

The fundus pathologies in the 200 eyes in the CKD group are summarized in Table 3. The most common finding was early AMD (20.5%). Diabetic retinopathy was present in 8% of the eyes. Table 4 compares the parafoveal vessel densities and foveal parameters between the control and CKD groups. Parafoveal vessel density was significantly decreased in the CKD group, in both SVP and DVP. This finding was consistent in all four parafoveal quadrants. Localized (Figure 3) or diffuse (Figure 4) rarefaction of retinal capillaries were observed in some patients with CKD. Other possible pathological changes of retinal capillary included blunt‐ended vessels, increased vascular tortuosity, and localized non‐perfusion area (Figure 3).

**Table 3:** Fundus pathologies in 200 eyes in CKD group

Fundus pathologies	n (%)
Early AMD	41 (20.5)
Diabetic retinopathy	16 (8)
Mild NPDR	5 (2.5)
Moderate NPDR	5 (2.5)
Severe NPDR	4 (2)
PDR	2 (1)
Hypertensive retinopathy	11 (5.5)
Epiretinal membrane	9 (4.5)
Asymptomatic retinal vein occlusion	2 (1)
Suspected hydroxychloroquine retinopathy	1 (0.5)

Abbreviations: NPDR, non‐proliferative diabetic retinopathy; PDR, proliferative diabetic retinopathy.

**Table 4:** Parafoveal vessel densities and foveal parameters in control group (50 eyes) and CKD group (n = 200 eyes)

	Control	CKD	*P* value
**SVP vessel density, % (mean ± SD, range)**			
Parafoveal	49.7 ± 2.9, 43.7‐55.8	46.7 ± 4.3, 34.2‐54.4	<0.001
Temporal	48.5 ± 2.8, 43.5‐53.6	45.4 ± 4.5, 32.1‐54.9	<0.001
Superior	50.8 ± 3.3, 43.5‐57.3	47.8 ± 4.6, 32.8‐56.0	<0.001
Nasal	49.1 ± 3.1, 42.8‐55.2	46.3 ± 4.3, 33.7‐54.6	<0.001
Inferior	50.6 ± 3.5, 41.8‐57.4	47.3 ± 4.8, 29.6‐55.9	<0.001
**DVP vessel density, % (mean ± SD, range)**			
Parafoveal	52.6 ± 2.9, 46.2‐58.2	50.1 ± 4.1, 37.9‐58.9	<0.001
Temporal	53.1 ± 2.9, 46.6‐59.6	50.2 ± 4.1, 37.0‐59.2	<0.001
Superior	52.4 ± 3.4, 44.2‐59.0	49.9 ± 4.5, 36.7‐59.9	<0.001
Nasal	53.4 ± 2.8, 47.4‐59.0	50.7 ± 4.2, 38.4‐60.0	<0.001
Inferior	51.7 ± 3.5, 44.0‐57.8	49.4 ± 4.6, 36.2‐59.3	<0.001
**Foveal parameters (mean ± SD, range)**			
FAZ size (mm^2^)	0.295 ± 0.101, 0.072‐0.522	0.327 ± 0.133, 0.017‐0.824	0.118
FAZ perimeter (mm)	2.155 ± 0.419, 1.039‐3.257	2.296 ± 0.533, 1.169‐3.724	0.085
FAZ a‐circularity index	1.14 ± 0.04, 1.08‐1.27	1.16 ± 0.07, 1.07‐1.52	0.002
FD‐300 (%)	49.9 ± 4.1, 38.0‐56.7	47.6 ± 4.6, 30.4‐56.6	0.001

**Figure 5:**
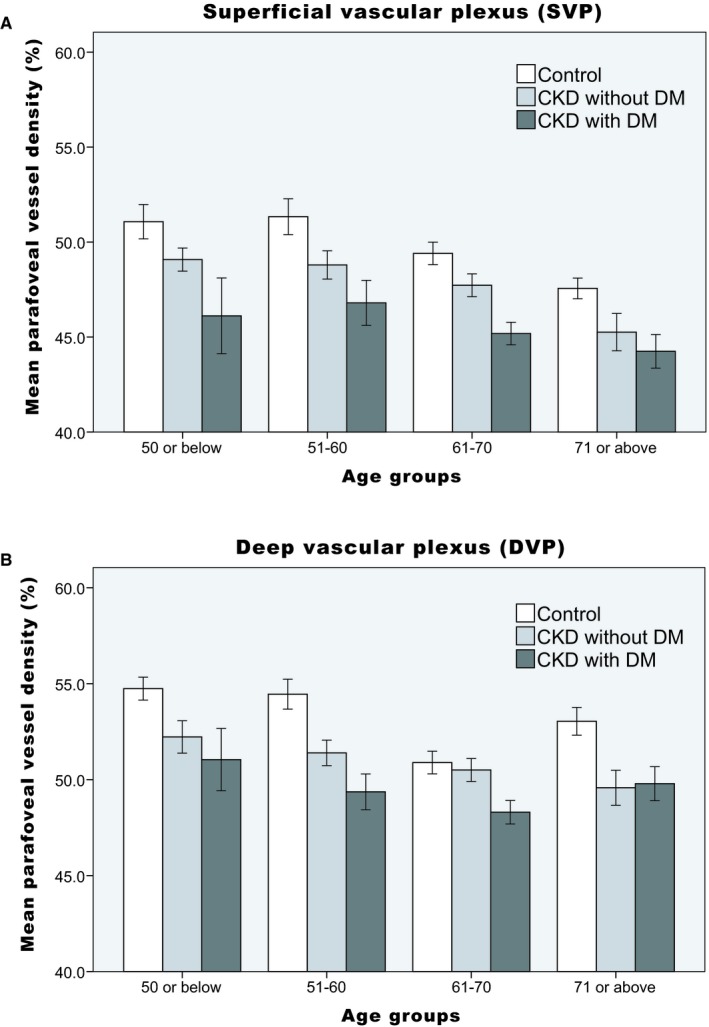
The distribution of mean parafoveal vessel density in (A) the SVP and (B) the DVP. Error bars depict the 95% confidence interval

Table 5 shows the multiple linear regression models for SVP and DVP vessel densities in all subjects (Model 1) and in patients with CKD (Model 2). In Model 1, age, DM, and CKD were negatively associated with both SVP and DVP vessel densities. Use of anti‐hypertensive drugs was positively associated with SVP vessel density, while smoking was negatively associated with DVP vessel density. In Model 2, age and DM were negatively associated with both SVP and DVP vessel densities. eGFR and use of anti‐hypertensive drugs were positively associated with SVP vessel density; smoking was negatively associated with DVP vessel density. Figure 5 demonstrates the distribution of mean parafoveal vessel density in the control, CKD without DM, and CKD with DM groups among different age‐groups. A trend toward vessel density reduction with aging and the presence of DM was observed in both SVP and DVP.

**Table 5:** Multiple linear regression models for vessel densities

	Parafoveal SVP vessel density			Parafoveal DVP vessel density		
	Coefficient	95% CI	*P* value	Coefficient	95% CI	*P* value
**Model 1 (multiple linear regression model for vessel densities in all 250 subjects)**						
Age	−0.116	−0.164 to −0.069	<0.001	−0.076	−0.124 to −0.028	0.002
DM	−2.068	−3.133 to −1.003	<0.001	−1.512	−2.571 to −0.454	0.005
CKD	−3.544	−5.431 to −1.656	<0.001	−1.953	−3.247 to −0.660	0.003
Use of anti‐hypertensive drug(s)	2.081	0.500 to 3.663	0.010			
Smoking				−0.755	−1.454 to −0.056	0.034
**Model 2 (multiple linear regression model for vessel densities in 200 CKD patients)**						
Age	−0.121	−0.176 to −0.066	<0.001	−0.067	−0.122 to −0.012	0.017
DM	−2.227	−3.352 to −1.101	<0.001	−1.541	−2.658 to −0.424	0.007
eGFR	0.030	0.002 to 0.058	0.036			
Use of anti‐hypertensive drug(s)	2.084	0.436 to 3.733	0.013			
Smoking				−0.806	−1.607 to −0.005	0.049

*Note*

Model 1: Multiple linear regression model with backward stepwise method in all 250 subjects: age, sex, smoking, BMI, DM, use of anti‐hypertensive drug(s), systolic BP, diastolic BP, and CKD were independent variables entered into the models.

Model 2: Multiple linear regression model with backward stepwise method in 200 patients with CKD: age, sex, smoking, BMI, DM, use of anti‐hypertensive drug(s), systolic BP, diastolic BP, CKD stage, and eGFR were independent variables entered into the models.

Abbreviation: CI, confidence interval.

## 4 DISCUSSION

There were two major findings in this study. First, retinal microvascular alterations may occur early in patients with CKD, before the onset of visual symptoms. Secondly, the microvasculature in different retinal layers may respond differently to systemic comorbidities.

Chronic kidney disease has been associated with increased visual impairment and ocular diseases in prior epidemiology studies.^10^, ^11^, ^12^ In current study, the BCVA in the CKD group was worse than that of the control group. There was also a trend toward visual acuity decreasing with increased CKD severity (Figure 2). We found a high prevalence of early AMD (20.5%) among patients with CKD. Other retinopathies were not very common in this study because we enrolled visually asymptomatic patients. Our results represent the early retinal microvascular changes in patients with CKD.

We found significant retinal microvascular abnormalities in patients with CKD. The quantitative changes included capillary rarefaction in both SVP and DVP, decreased FD‐300 vessel density, and increased a‐circularity index of FAZ. Multiple regression models (Model 1) showed that CKD is an independent factor associated with decreased vessel densities in both SVP and DVP. Decreased vessel density may result from the localized or diffuse rarefaction of capillaries (Figure 3). Increased a‐circularity index of FAZ may be caused by the disruption of parafoveal capillary networks (Figure 4). Optical coherence tomography angiography also enabled us to visualize the morphological changes at the capillary level in these patients, such as blunt‐ended vessels, increased vascular tortuosity, and localized non‐perfusion area (Figure 3). The severity of retinal microvascular alteration may vary among patients with CKD. The multiple regression models (Model 2) illustrated the associated systemic factors in patients with CKD.

Age and DM are important factors negatively associated with vessel density in both SVP and DVP. A prior OCTA study has demonstrated that aging is associated with decreased vessel density in both the superficial and deep capillary plexus in the normal population.^26^ Chronic kidney disease may also contribute to premature aging of microcirculation.^27^ Diabetes mellitus was present in 91 (45.5%) patients in the current study. It had been well known that the reduction of vessel density is correlated to the severity of diabetic retinopathy.^28^, ^29^ Although most of our patients did not have any diabetic retinopathy, prior OCTA studies had demonstrated that microvascular changes may occur before clinically detectable diabetic retinopathy.^30^, ^31^, ^32^, ^33^, ^34^

Hypertension is very common among patients with CKD. About 85% of patients with CKD may have coexistent hypertension.^35^ In our study, the most commonly used class of anti‐hypertensive drug was ACEI/ARB (73.1%), followed by calcium channel blocker (49.7%). Prior studies showed that both ACEI/ARB and calcium channel blockers may improve the retinal arteriolar narrowing and capillary rarefaction in hypertensive patients.^36^, ^37^ The benefit of anti‐hypertensive drugs may have resulted either from better‐controlled BP^38^ or from other pharmacological effects independent of the BP lowering effect. For an example, a localized RAS had been found within the eye, such as in retinal microvasculature, Müller cells, and ganglion cells.^39^ The activation of RAS may promote retinal neovascularization, inflammation, oxidative stress, and neuronal and glial dysfunction.^39^ So, one of the possible mechanisms may be a protective effect in the retina via suppression of RAS by ACEI/ARB.^39^

Cigarette smoking is a common risk factor for CKD and various ocular diseases such as AMD and cataract.^11^ It has been well known that cigarette smoking can result in morphological changes (ie, vessel wall injury, capillary loss) and functional changes in microcirculation.^40^ Interestingly, smoking was negatively associated with vessel density in DVP, but not in SVP, in the current study. A similar finding has also been reported previously in diabetic patients without diabetic retinopathy.^41^ Current smoker status was correlated with lower vessel density in the deep capillary plexus, but not associated with vessel density in the superficial capillary plexus.^41^ Further study is necessary to confirm this observation and to determine why deep retinal capillaries are more susceptible to injury from cigarette smoking.

In our study, vessel densities in SVP and DVP were associated with different systemic factors. There are three capillary plexuses over the parafoveal area, namely the superficial, intermediate, and deep capillary plexus.^42^ In vivo human study showed that each of these three capillary plexuses may have its own feeding arteriolar supply and draining venules.^43^ Each capillary plexus has different anatomical structures and may have its own autoregulation.^43^ The plexuses may respond differently to systemic condition alteration, such as changes in BP and oxygenation, or to retinal functional hyperemia evoked by a flickering light stimulus.^44^, ^45^, ^46^ In our study, anti‐hypertensive drugs and eGFR were associated with vessel density in SVP, but not in DVP. On the contrary, smoking was associated with vessel density in DVP, but not in SVP. Therefore, our results support the hypothesis that microvasculature in different retinal layers may respond differently to varying systemic factors.

There are several limitations in this study. The study is limited by its small sample size and cross‐sectional study design. Longitudinal follow‐up data were not available. Furthermore, we enrolled patients without visual symptoms into the CKD group. So, our results may reflect early retinal microvascular alterations rather than late‐stage retinopathies.

In summary, our study demonstrated that patients with CKD had significant rarefaction of retinal microvasculature in both SVP and DVP. Morphological changes in the retinal capillaries were observed via OCTA. The microvasculature in the different retinal layers may respond differently to varying systemic factors. Ophthalmologists should take these microvascular changes into consideration when interpreting OCTA images in patients with CKD.

## PERSPECTIVE

Optical coherence tomography angiography showed that patients with CKD may have rarefaction and morphological changes of retinal microvasculature in the superficial and DVPs. The microvasculature in different retinal layers may respond differently to various systemic factors.

## CONFLICT OF INTEREST

No authors have any financial/conflicts of interest to disclose.
